# Socioeconomic factors and non-admission to the intensive care unit decisions in hospitalised COVID-19 patients: a retrospective cohort study in Stockholm, Sweden

**DOI:** 10.1136/bmjopen-2026-120447

**Published:** 2026-08-27

**Authors:** Adonis Sotoodeh, Pontus Hedberg, Johan Zetterqvist, Johan Mårtensson, Pontus Naucler

**Affiliations:** 1 Division of Infectious Diseases, Karolinska Institute Department of Medicine Solna, Stockholm, Sweden; 2 Department of Medicine, Huddinge, Karolinska Institute, Stockholm, Sweden; 3 Karolinska Institute Clinical Epidemiology Division, Stockholm, Sweden; 4 Karolinska Institute Department of Physiology and Pharmacology, Stockholm, Sweden; 5 Karolinska University Hospital Department of Infectious Diseases, Stockholm, Sweden

**Keywords:** Health Equity, PUBLIC HEALTH, INTENSIVE & CRITICAL CARE, Public health, COVID-19

## Abstract

**Abstract:**

**Objective:**

To investigate the association between socioeconomic factors (education level, region of birth and household composition) and non-admission to the intensive care unit (ICU) decisions in patients hospitalised with COVID-19.

**Design:**

Retrospective multicentre cohort study using linkage of several population-based health registries.

**Setting:**

Five acute care hospitals in Region Stockholm, Sweden, between March 2020 and February 2023.

**Participants:**

Data were extracted from 20 764 patients aged ≥18 years hospitalised with COVID-19.

**Main outcome measure:**

A recorded decision of non-admission to the ICU within 72 hours of admission. We used a modified Poisson regression model for each socioeconomic factor separately, adjusting for the other socioeconomic factors, age, sex, comorbidities, care services, calendar time, hospital and ICU occupancy.

**Results:**

Among 20 764 individuals, 2823 (13.6%) received a non-admission to the ICU decision. In the adjusted model, completion of primary or secondary education as the highest level was associated with an increased risk of receiving a decision of non-admission to the ICU (adjusted risk ratio (aRR) 1.20 (95% CI 1.10 to 1.30, p<0.01) and aRR 1.14 (95% CI 1.05 to 1.23, p<0.01), respectively) compared with completion of tertiary education. Being born in Northwestern Europe and Northern America, as opposed to Sweden specifically, was also associated with an increased risk (aRR 1.15 (95% CI 1.06 to 1.26, p<0.01)).

**Conclusion:**

We observed an increased risk of receiving a non-admission to the ICU decision for patients with lower levels of education and those born in Northwestern Europe or Northern America. This calls for action by physicians and policymakers to address socioeconomic inequities in access to intensive care.

**Strengths and limitations of this study**

- Individual-level linkage of data across multiple registries enabled us to account for a comprehensive range of factors, including comorbidities documented in both primary and specialist care, electronic health record data, nursing home residency, hospital and intensive care unit (ICU) occupancy, thereby strengthening the validity and relevance of our findings.
- This is a multicentre study, which reduces the risk of systematic bias within a hospital or socioeconomic differences in the uptake area.
- The study period spans 3 years of the pandemic, allowing the results to be analysed over time.
- There may be selection bias, as patients had to be hospitalised to be included in this study. Different healthcare regulations were implemented throughout the pandemic, with the elderly population and nursing home residents experiencing greater difficulties in accessing inpatient care during the first wave.
- We could not examine the underlying medical indication for a patient to receive a decision of non-admission to the ICU.

## Introduction

 The intensive care unit (ICU) provides the highest level of care with the possibility of intensive medical and nursing care.^1^ During the COVID-19 pandemic, a considerable number of patients with severe COVID-19 required treatment in the ICU due to respiratory failure. However, withholding such treatment should be considered if a patient refuses treatment or if treatment is deemed futile.^2^ In Sweden, the decision to withhold ICU care requires a comprehensive assessment by at least two licensed healthcare professionals, one of whom must be a licensed physician.^2^ In addition, the physician responsible for the patient’s care must thoroughly document the decision according to the Health and Medical Services Act.^2^

Previous research has shown that the decision to withhold or withdraw life-sustaining treatment is more common in patients with greater illness severity, multiple organ failure and severe comorbidities.^3^ In addition, we have previously reported an increased risk of non-admission to the ICU decisions during periods of higher occupancy, particularly when ICU occupancy by COVID-19 patients was elevated.^4^ However, limited research exists on the influence of socioeconomic factors on decisions of non-admission to the ICU. Socioeconomic factors affect health-seeking behaviour, with lower socioeconomic status associated with greater illness severity at the time of seeking care and lower health literacy; factors that may contribute to an increased risk of receiving a decision of non-admission to the ICU.^5^ A national Swedish study covering the period 2014–2020 reported a positive association between decisions to withhold or withdraw any life or organ support and lower levels of education, lower income and for individuals living in a single household. However, these findings were restricted to patients already admitted to the ICU, which may not reflect patterns in the total hospital population.^6^

We aimed to investigate the association between socioeconomic factors (education level, region of birth and household composition) and non-admission to the ICU decisions among all patients hospitalised with COVID-19 who were admitted through the emergency department.

## Methods

### Study design and patient population

We performed a retrospective multicentre cohort study of all hospitalised adult patients (aged ≥18 years) with COVID-19 admitted to five acute care hospitals in Region Stockholm (Karolinska University Hospital (Solna and Huddinge sites), Danderyd Hospital, Södersjukhuset, Södertälje Hospital and Norrtälje Hospital) between 1 March 2020 and 28 February 2023. Patients were classified as hospitalised with COVID-19 if they received inpatient treatment with a diagnosis of COVID-19, according to the International Classification of Diseases and Related Health Problems, Tenth Revision (ICD-10) codes U07.1 and U07.2, and had a positive SARS-CoV-2 test recorded in SmiNet within a window from 14 days prior to 3 days after hospital admission. Only patients admitted through the emergency department were included, corresponding to approximately 94% of those who otherwise met the inclusion criteria. Patients who had not resided in the region for at least 3 years prior to the date of admission were excluded to ensure adequate baseline characteristics. For patients with multiple hospitalisations during the pandemic, only the first healthcare episode with COVID-19 was retained.

### Data sources and linkages

In Sweden, each person in the Swedish Population Register is assigned a unique personal identity number, which facilitates linkage between population-based health registries for research purposes.^7^ In our study, we linked data from: the Intelligence Data Warehouse of electronic health records, Stockholm Regional Healthcare Data Warehouse (VAL), the Swedish Intensive Care Registry, SmiNet and Statistics Sweden. The Intelligence Data Warehouse provided data on all treatment limitations, including decisions of non-admission to the ICU and disease severity. VAL, operated by the Stockholm Regional Council, contains comprehensive data on comorbid conditions, inpatient visits, outpatient visits, care services and covers approximately 94% of all primary care visits in Region Stockholm.^8^ Occupancy data were acquired from the Swedish Intensive Care Registry. SmiNet is an electronic system operated by the Public Health Agency of Sweden and communicable disease control units, used for the surveillance of notifiable infectious diseases, including COVID-19, in accordance with the Communicable Diseases Act and the Communicable Diseases Ordinance.^9^ Data on all positive PCR tests for SARS-CoV-2 were obtained from SmiNet. Socioeconomic data on education level, region of birth and household composition were obtained from the Longitudinal Integration Database for Health Insurance and Labour Market Research (LISA) from Statistics Sweden.

### Variable definitions

Education level was categorised as primary (≤9 years), secondary (>9 and ≤12 years) and tertiary (>12 years) education. Region of birth was based on a modified version of the United Nations’ geoscheme and grouped into eight categories: Sweden, Northwestern Europe and Northern America, Southern Europe, Eastern Europe, Western and Central Asia, East Asia and Oceania, Africa, and Latin America and the Caribbean. Household composition was dichotomised into single-person and multi-person households.

Comorbidities were derived from ICD-10 codes for diseases previously recognised as associated with an increased risk of severe COVID-19 in accordance with the National Board of Health and Welfare in Sweden and the Centers for Disease Control and Prevention.^10 11^ Restricted cubic splines were used to model calendar time. Knots were placed at: the publication of the dexamethasone results from the RECOVERY trial (1 July 2020), the emergence of the alpha variant in Sweden (15 February 2021), the roll-out of the second COVID-19 vaccine dose for adults aged 70 years and older (1 July 2021) and the period following the Omicron variant surge in Europe (9 March 2022). ICU occupancy was assessed using a 7-day rolling average number of ICU patients with COVID-19 for each calendar day, including the date of admission, capturing fluctuations in ICU pressure across the study period, including pandemic peaks. Care services were defined as residence in a nursing home, home care services and advanced medical care at home. Disease severity was assessed using the National Early Warning Score (NEWS), which includes respiratory rate, oxygen saturation, oxygen therapy (including nasal cannula, face mask, high-flow nasal cannula and non-invasive ventilation), systolic blood pressure, pulse, consciousness and body temperature.^12^ The highest NEWS score recorded up to 24 hours after admission was used, including pre-admission values from the emergency department.

### Outcomes

The outcome of the study was a recorded decision of non-admission to the ICU or coronary ICU within 72 hours of admission. If multiple decisions of non-admission to the ICU were made, the most restrictive decision was recorded. In the absence of a recorded decision of any treatment limitation, the main model assumed that no treatment limitations applied, reflecting routine clinical practice in such cases. In a sensitivity analysis, the study outcome was imputed when no decision was recorded.

### Statistical analysis

Patients were grouped based on the presence of a non-admission to the ICU decision. Baseline characteristics were presented using descriptive statistics with categorical variables as counts with percentages and numeric values as medians with IQRs. Three variables (education level, region of birth and household composition) had limited missing data, for which complete-case analyses were performed in the main analysis.

Modified Poisson regression models were used to examine the risk of non-admission to the ICU decisions for each of the three socioeconomic variables, adjusted for the other socioeconomic variables, age, sex, comorbidities, care services, calendar time, hospital and ICU occupancy. We then performed five predefined sensitivity analyses: (1) restriction to patients with a COVID-19 diagnosis code as the main diagnosis, (2) non-admission to the ICU decision also including a combination of non-resuscitation limitation and mechanical ventilation limitation as the study outcome, (3) last decision of non-admission to the ICU within the first 72 hours after admission, (4) application of multiple imputation by chained equation (MICE) for socioeconomic variables, and (5) application of MICE for non-admission to the ICU decisions. A detailed description of multiple imputation in the statistical analyses is provided in the electronic online supplemental (ESM) methods. Additionally, we conducted two subgroup analyses by restricting the study period separately to the first wave and to the subsequent waves. Finally, we tested for interaction between the socioeconomic variables and the dichotomous variables NEWS ≥7 and age ≥75 years, as well as calendar time (modelled using restricted cubic splines), using Wald tests. All analyses were performed with R V.4.1.0 (R Foundation for Statistical Computing, Vienna, Austria).

### Patient and public involvement

This study was inspired by the clinical experience of AS, JM and PN. Patients and members of the public were not directly involved, as the research consisted of a retrospective analysis of restricted-access, population-based data sources.

## Results

**Figure 1:**
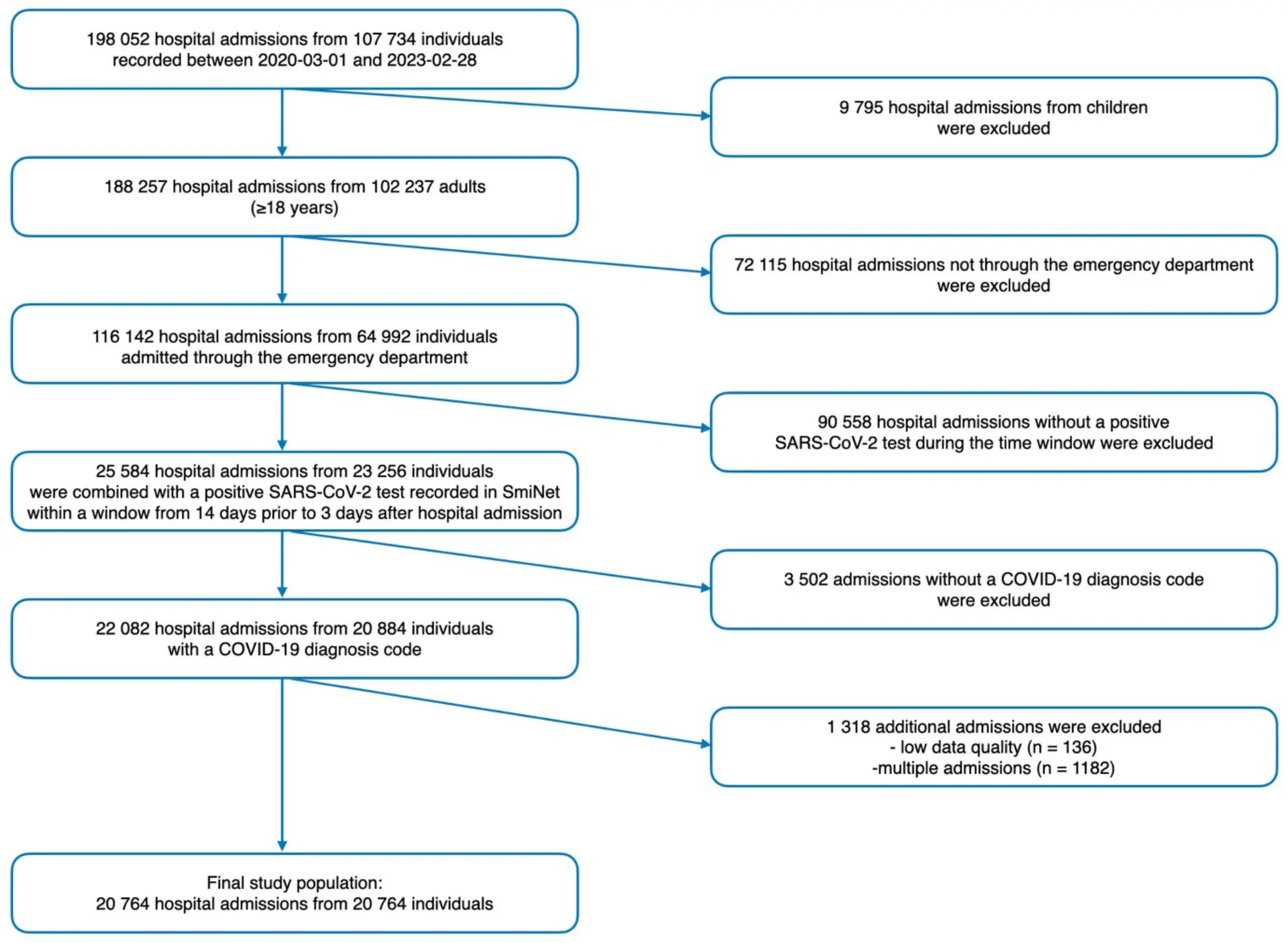
Flowchart of hospital admissions and individuals included in this study.

A total of 198 052 hospital admissions from 107 734 individuals were registered in the included acute hospitals between March 2020 and February 2023, of which 20 764 individuals were considered due to COVID-19 and did not fulfil any of the exclusion criteria (figure 1). Out of the 20 764 individuals included in this study, 2823 (13.6%) received a decision of non-admission to the ICU. Patients with a non-admission to the ICU decision were older and more often female than the group without a non-admission to the ICU decision (48.7% vs 40.4%; table 1). The prevalence of comorbidities and care services was higher in the group with a non-admission decision. Individuals in the non-admission group were more likely to have been born in Sweden, 1958 (69.4%) patients, compared with the group without a non-admission decision, 10 187 (56.8%) patients. Completion of tertiary education was less common in the non-admission group, 584 (20.7%) individuals, than in the group without a non-admission decision, 5578 (31.1%) individuals. A larger proportion of patients in the non-admission group lived in a single-person household (1460 (51.7%) compared with 5060 (28.2%) patients). A stratified analysis, including the outcome categories: recorded decision with any treatment limitation, recorded decision without any treatment limitations and absence of a recorded decision, is presented in the electronic online supplemental ESM table 1.

**Table 1:** Baseline characteristics of study participants stratified by non-admission to the ICU decision

	Overall n=20 764	Decision of non-admission to the ICU n=2823	No decision of non-admission to the ICU n=17 941
Age group			
18–49 years	3979 (19.2)	17 (0.6)	3962 (22.1)
50–59 years	3310 (15.9)	43 (1.5)	3267 (18.2)
60–69 years	3726 (17.9)	146 (5.2)	3580 (20.0)
70–79 years	4550 (21.9)	621 (22.0)	3929 (21.9)
80–89 years	3838 (18.5)	1226 (43.4)	2612 (14.6)
≥90 years	1361 (6.6)	770 (27.3)	591 (3.3)
Sex			
Female	8615 (41.5)	1374 (48.7)	7241 (40.4)
Male	12 149 (58.5)	1449 (51.3)	10 700 (59.6)
Comorbidities			
Asthma	2352 (11.3)	262 (9.3)	2090 (11.6)
Cancer	1741 (8.4)	356 (12.6)	1385 (7.7)
Cardiac/cerebrovascular	6571 (31.6)	1760 (62.3)	4811 (26.8)
Chronic kidney disease	2416 (11.6)	767 (27.2)	1649 (9.2)
Chronic liver disease	557 (2.7)	77 (2.7)	480 (2.7)
Chronic lung disease	2390 (11.5)	546 (19.3)	1844 (10.3)
Diabetes	4895 (23.6)	858 (30.4)	4037 (22.5)
Hypertension	10 038 (48.3)	2045 (72.4)	7993 (44.6)
Immunosuppression	2694 (13.0)	409 (14.5)	2285 (12.7)
Neurology/Dementia	1833 (8.8)	766 (27.1)	1067 (5.9)
Obesity	2286 (11.0)	195 (6.9)	2091 (11.7)
Psychiatric	5355 (25.8)	727 (25.8)	4628 (25.8)
Care services	4524 (21.8)	1843 (65.3)	2681 (14.9)
First wave	4967 (23.9)	1075 (38.1)	3892 (21.7)
NEWS, median (IQR), points	4 (2–6)	4 (2–6)	6 (3–8)
Education level			
Primary education	5261 (25.3)	981 (34.8)	4280 (23.9)
Secondary education	8356 (40.2)	1065 (37.7)	7291 (40.6)
Tertiary education	6162 (29.7)	584 (20.7)	5578 (31.1)
Missing	985 (4.7)	193 (6.8)	792 (4.4)
Region of birth			
Sweden	12 145 (58.5)	1958 (69.4)	10 187 (56.8)
Northern Europe	1307 (6.3)	300 (10.6)	1007 (5.6)
Western Europe	259 (1.2)	69 (2.4)	190 (1.1)
Southern Europe	773 (3.7)	89 (3.2)	684 (3.8)
Eastern Europe	657 (3.2)	63 (2.2)	594 (3.3)
Western and Central Asia	2680 (12.9)	206 (7.3)	2474 (13.8)
East Asia and Oceania	1243 (6.0)	57 (2.0)	1186 (6.6)
Northern Africa	279 (1.3)	9 (0.3)	270 (1.5)
Sub-Saharan Africa	586 (2.8)	34 (1.2)	552 (3.1)
Latin America and the Caribbean	703 (3.4)	32 (1.1)	671 (3.7)
Northern America	57 (0.3)	1 (0.0)	56 (0.3)
Missing	75 (0.4)	5 (0.2)	70 (0.4)
Household composition			
Single-person household	6520 (31.4)	1460 (51.7)	5060 (28.2)
Multi-person household	14 036 (67.6)	1347 (47.7)	12 689 (70.7)
Missing	208 (1.0)	16 (0.6)	192 (1.1)

Note: values are shown as counts with percentages representing proportions in parenthesis.

ICU, intensive care unit; NEWS, national early warning score.

### Adjusted analyses of non-admission to the ICU decision

**Figure 2:**
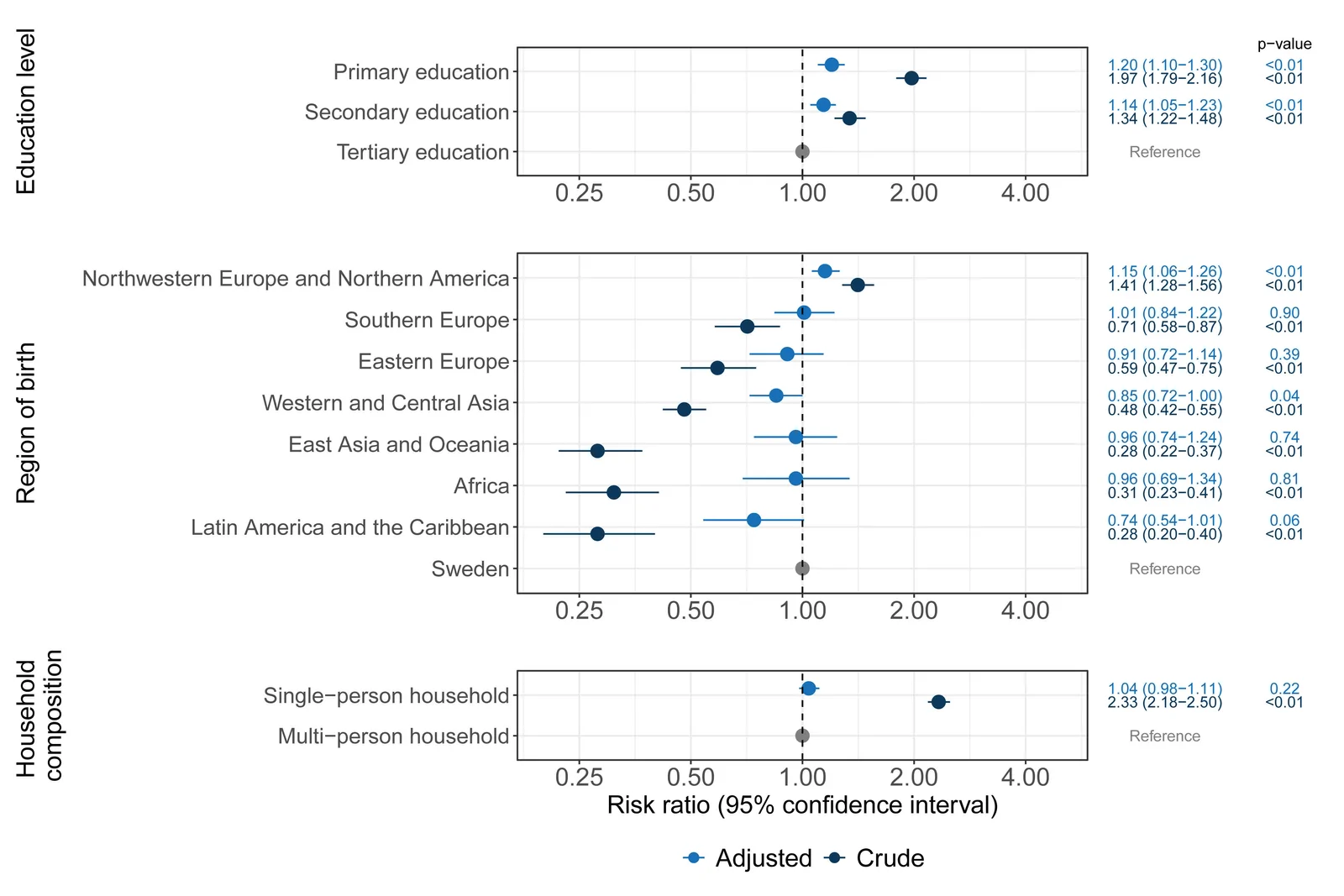
Adjusted risk ratios for non-admission to the ICU. Importantly, the three multivariable models included the socioeconomic variable of interest (education level, region of birth and household composition) adjusted for the other socioeconomic factors, age, sex, comorbidities, care services, calendar time, hospital and ICU occupancy. ICU, intensive care unit.

In the multivariable regression model, completion of primary or secondary education as the highest level of education was associated with an increased risk of receiving a non-admission to the ICU decision (adjusted risk ratio (aRR) 1.20 (95% CI 1.10 to 1.30, p<0.01) and aRR 1.14 (95% CI 1.05 to 1.23, p<0.01), respectively), compared with completion of tertiary education (figure 2). Being born in Northwestern Europe and Northern America, as opposed to Sweden specifically, was also associated with an increased risk (aRR 1.15 (95% CI 1.06 to 1.26), p<0.01). No statistically significant association was found for individuals living in single-person households compared with individuals living in multi-person households (aRR 1.04 (95% CI 0.98 to 1.11), p=0.22).

The results were similar in sensitivity analyses with a restriction to COVID-19 as the main diagnosis, application of a broader definition of non-admission to the ICU, a restriction to the last decision of non-admission to the ICU within the first 72 hours from admission and application of multiple imputation (table 2). Subgroup analyses with restrictions of the study period to the first wave and subsequent waves, respectively, showed comparable results. In interaction analyses, interactions were found between household composition with NEWS ≥7 points (p=0.0009), education level with age ≥75 years (p=0.0098), household composition with age ≥75 years (p = <0.0001), region of birth with calendar time (p=0.0010), and household composition with calendar time (p=0.0004; online supplemental ESM table 2). Stratified analyses for the categorical effect modifiers (age≥75 years and NEWS≥7 points) are provided in the online supplemental ESM figure 1). As calendar time was modelled using restricted cubic splines, the corresponding interactions could not be summarised as stratum-specific ORs and are therefore not presented as stratified plots.

**Table 2:** Sensitivity and subgroup analyses

	Main analysis	COVID-19 as the main diagnosis	Broad definition of non-admission to the ICU	Last decision of non-admission to the ICU	Multiple imputation of socioeconomic variables	Multiple imputation of non-admission to the ICU decisions	Study period restricted to the first wave	Study period from the second wave onwards
Education level								
Primary education	1.20 (1.10–1.30)	1.17 (1.07–1.28)	1.18 (1.10–1.26)	1.18 (1.09–1.29)	1.20 (1.08–1.33)	1.15 (1.03–1.28)	1.21 (1.08–1.35)	1.18 (1.05–1.31)
Secondary education	1.14 (1.05–1.23)	1.13 (1.03–1.23)	1.14 (1.06–1.22)	1.14 (1.05–1.23)	1.14 (1.02–1.26)	1.08 (0.97–1.20)	1.11 (1.00–1.24)	1.13 (1.01–1.26)
Tertiary education	Reference	Reference	Reference	Reference	Reference	Reference	Reference	Reference
Region of birth								
Northwestern Europe and Northern America	1.15 (1.06–1.26)	1.07 (0.97–1.18)	1.14 (1.06–1.23)	1.15 (1.05–1.25)	1.15 (1.03–1.29)	1.09 (0.98–1.21)	1.06 (0.94–1.19)	1.21 (1.09–1.36)
Southern Europe	1.01 (0.84–1.22)	1.05 (0.86–1.29)	1.04 (0.89–1.23)	1.02 (0.84–1.23)	1.01 (0.80–1.28)	1.04 (0.83–1.31)	0.94 (0.74–1.19)	1.03 (0.79–1.35)
Eastern Europe	0.91 (0.72–1.14)	0.97 (0.77–1.22)	0.89 (0.72–1.09)	0.92 (0.74–1.16)	0.91 (0.68–1.21)	0.93 (0.71–1.23)	0.78 (0.54–1.13)	0.95 (0.72–1.26)
Western and Central Asia	0.85 (0.72–1.00)	0.83 (0.70–0.98)	0.84 (0.73–0.97)	0.86 (0.73–1.01)	0.85 (0.70–1.03)	0.90 (0.74–1.09)	0.76 (0.63–0.92)	0.88 (0.67–1.16)
East Asia and Oceania	0.96 (0.74–1.24)	0.84 (0.62–1.14)	0.82 (0.64–1.05)	0.92 (0.70–1.20)	0.96 (0.70–1.32)	1.01 (0.75–1.36)	0.96 (0.66–1.40)	0.98 (0.69–1.39)
Africa	0.96 (0.69–1.34)	1.03 (0.74–1.43)	0.87 (0.64–1.19)	0.90 (0.63–1.27)	0.96 (0.66–1.40)	1.08 (0.75–1.54)	1.26 (0.87–1.83)	0.59 (0.30–1.16)
Latin America and the Caribbean	0.74 (0.54–1.01)	0.75 (0.55–1.03)	0.70 (0.52–0.93)	0.77 (0.56–1.04)	0.74 (0.51–1.07)	0.84 (0.60–1.18)	0.48 (0.32–0.73)	1.13 (0.73–1.73)
Sweden	Reference	Reference	Reference	Reference	Reference	Reference	Reference	Reference
Household composition								
Single-person household	1.04 (0.98–1.11)	1.03 (0.96–1.10)	1.05 (0.99–1.11)	1.04 (0.98–1.11)	1.04 (0.96–1.13)	1.07 (0.98–1.16)	1.04 (0.95–1.14)	1.06 (0.97–1.15)
Multi-person household	Reference	Reference	Reference	Reference	Reference	Reference	Reference	Reference

Note: table 2 displays the adjusted risk ratios for each category of all socioeconomic variables in the main analysis along with five sensitivity analyses and two subgroup analyses: (1) COVID-19 as the main diagnosis, (2) broad definition of non-admission to the ICU also including the combination of a do-not-resuscitate order and limitation of invasive mechanical ventilator, (3) last decision of non-admission to the intensive care within the first 72 hours after admission, (4) application of multiple imputation of socioeconomic variables, (5) application of multiple imputation of non-admission to the ICU decisions, (6) study period restricted to the first wave and (7) study period from the second wave. All models included the socioeconomic variable of interest (education level, region of birth and household education), adjusted for the other socioeconomic factors, age, sex, comorbidities, care services, calendar time, hospital and ICU occupancy.

ICU, intensive care unit.

## Discussion

### Principal findings

In this register-based cohort study of 20 764 individuals, we observed an association between individual-level socioeconomic factors (education level, region of birth and household composition) and non-admission to the ICU decisions. In the adjusted analyses for each of the socioeconomic factors, the highest relative risk was associated with lower education level. An increased risk of receiving a non-admission to the ICU decision was also found in individuals born in Northwestern Europe and Northern America. No differences were found between individuals living in single-person households or multi-person households.

### Comparison with related studies

The risk of receiving a non-admission to the ICU decision increased gradually for patients whose highest level of completed education was secondary or primary education, compared with tertiary education. A Swedish national study among patients treated in the ICU previously showed an inverse association with a lower risk of a decision to withhold or withdraw life or organ support in patients with a university degree compared with those with upper secondary or lower secondary or lower education.^6^ Patient involvement in end-of-life decisions has been reported to be higher among those who have completed higher education.^13^ This could be due to patients with lower levels of education wanting to avoid these difficult decisions and opting to rely on the physician’s judgement.^13^ Alternatively, it could reflect differences in health literacy, as patients with higher education levels tend to communicate more actively and show greater emotional expressiveness, which might encourage more information exchange with doctors.^13^ Interestingly, a Belgian study reported that physicians consulted colleagues more frequently when making end-of-life decisions for patients with higher levels of education.^14^ One explanation could be that interactions with patients and their families lead physicians to consult colleagues, or perhaps the perception of a more educated patient encourages physicians to approach the decision-making process with extra caution.^14^

In addition, we show a higher risk of receiving a non-admission decision for patients born in Northwestern Europe and Northern America compared with patients born in Sweden. Also, a lower risk of receiving a non-admission decision was found in patients born in Western and Central Asia. A similar pattern was reported by Strandberg *et al*, although analysed by continental regions.^6^ We have included more specific regions here and can show differences even within continents. Evidently, there are differences in the way different cultures deal with end-of-life-issues. A survey conducted in Europe showed large differences in decisions to withhold or withdraw care, with the frequency of such decisions decreasing from Northern and Western Europe to Southern Europe.^15^ Such variation may be influenced by broader cultural and religious factors, including the religious beliefs of both physicians and patients which can influence end-of-life-decisions.^16^ Moreover, the role that the family plays in the decision to limit care differs from region to region; in North America, family involvement is common, whereas in the Asia-Pacific region, families may take decisions without the patients themselves, while in Europe, end-of-life-decisions are often made without involvement of any family members.^17^ Unfortunately, we did not have access to individual religious beliefs or reliable data on whether non-admission to the ICU decisions were made jointly together with family members.

We found no association between household composition and the risk of a non-admission to the ICU decision in the adjusted analyses, which was consistent across all sensitivity and subgroup analyses, although a substantial difference was observed in the crude model (figure 1). Our results are in contrast with the study by Strandberg *et al*, which showed that household composition is an independent risk factor. One explanation for the difference could be that data from the VAL database allow us to also capture comorbidities in primary care and data on care services (residence in a nursing home, home care services and advanced medical care at home), which are important factors to consider. Notably, household composition showed significant interactions with NEWS ≥7, age ≥75 years and calendar time, suggesting that its effect may not be uniform across time periods or patient severity and age groups, which could further contribute to the absence of a consistent overall association.

### Strengths and limitations

A key strength of our study lies in its inclusion of all hospitalised patients and thus extending the generalisability of our findings beyond the ICU-specific population examined in the study by Strandberg *et al*.^6^ Furthermore, this is a multicentre study, which reduces the risk of systematic bias within a hospital or socioeconomic differences in the uptake-area. In addition, the study period spans 3 years of the pandemic, which allows the results to be analysed over time. Moreover, the study cohort includes 20 764 patients, which allows for high statistical precision. Finally, the unique personal identity code in Sweden enables the linkage of different databases with high resolution and accuracy. Nevertheless, the study has several limitations. First, there could be selection bias as patients had to be hospitalised to be included in this study. Different healthcare regulations were implemented throughout the pandemic, with the elderly population and nursing home residents experiencing greater difficulties in accessing inpatient care during the first wave. Second, the analyses cover only the first COVID-19 related healthcare episode. Therefore, conclusions can be drawn only for the first stay in healthcare during the pandemic. Third, the categorisation of region of birth is coarse and may not fully capture significant cultural differences. Due to small sample sizes in certain regions, we were unable to explore these differences in detail. Fourth, our cohort began at hospital admission, so we could not determine whether socioeconomic factors influenced presentation to hospital from the community. While we would not anticipate socioeconomic influences on the emergency department’s clinical admission decision, any patterning in care-seeking behaviour, if present, would be expected to weaken rather than inflate the observed associations. Moreover, adjustments for severity of comorbidity diagnoses were not considered, for example, different stages of chronic kidney disease. Also, we could not examine the underlying medical indication for the patient to receive a decision of non-admission to the ICU.

## Conclusion

Our observations reveal an association between socioeconomic factors and increased risk of receiving a non-admission to the ICU decision; in particular, the risk was elevated among patients with a lower level of education. These results provide evidence that should be considered by policymakers and physicians to implement measures such as standardisation strategies for non-admission to the ICU decisions to address socioeconomic inequities in access to intensive care.

## Supplementary material

10.1136/bmjopen-2026-120447 online supplemental file 1

## Data Availability

The individual participant data underlying this article were subject to ethical approval and cannot be shared publicly. Data from the deidentified administrative health registry are not freely available due to the protection of the personal integrity of the participants.
