# Supplementary material for: Socioeconomic factors and non-admission to the intensive care unit decisions in hospitalised COVID-19 patients: a retrospective cohort study in Stockholm, Sweden
Source: BMJ Open. 2026 Aug 27;16(8):e120447. doi: 10.1136/bmjopen-2026-120447 (PMC13536059; doi:10.1136/bmjopen-2026-120447)
Supplement: Supplementary file 1 [file bmjopen-16-8-s001.pdf]

Electronic supplementary material

This supplement contains additional tables and figures to the study by, Adonis Sotoodeh, Pontus Hedberg, Johan Zetterqvist, Johan Mårtensson, and Pontus Nauclér:

"Socioeconomic factors and non-admission to the intensive care unit decisions in hospitalised COVID-19 patients: a retrospective cohort study"

Table of contents

ESM Methods	Detailed description of multiple imputation in statistical analyses (page 2)
ESM Table 1	Baseline characteristics of study participants stratified by recorded decisions of treatment limitations (page 3)
ESM Table 2	Interaction tests of socioeconomic factors with NEWS, age ≥75 years, and calendar time (page 4)
ESM Figure 1	Stratified analyses following identified interactions between socioeconomic factors and NEWS and age ≥75 years (page 5)

ESM Methods. Detailed description of multiple imputation in statistical analyses

Imputation of socioeconomic variables

Of the 20 764 individuals included in this study, 985 (4.7%) were missing data on education, 75 (0.4%) were missing data on region of birth, 208 (1.0%) were missing data on household composition. Categorical imputation was used to impute data on those using the other socioeconomic factors, age, sex, comorbidities, care services, calendar time, hospital and ICU occupancy. Complete data were available for all predictor variables except socioeconomic variables. Fifty imputated datasets (m = 50) were created with 5 iterations (maxit = 5) per imputation. The final estimates were obtained using Rubin's rules.

Imputation of the decision of non-admission to the intensive care unit

Of the 20 764 individuals included in this study, there was an absence of a recorded decision for 12 592 individuals (60.4%). Binary categorical imputation was used to impute data on those using the other socioeconomic factors, age, sex, comorbidities, care services, calendar time, hospital and ICU occupancy. Complete data were available for all predictor variables except socioeconomic variables. Fifty imputed datasets (m = 50) were created with 5 iterations (maxit = 5) per imputation. The final estimates were obtained using Rubin's rules.

ESM Table 1. Baseline characteristics of study participants stratified by recorded decisions of treatment limitations

	Recorded decision with any treatment limitation n = 4275	Recorded decision without any treatment limitations n = 3897	Absence of a recorded decision n = 12 592
Age group			
18-49 years	41 (1.0)	431 (11.1)	3507 (27.9)
50-59 years	68 (1.6)	590 (15.1)	2652 (21.1)
60-69 years	244 (5.7)	871 (22.4)	2611 (20.7)
70-79 years	960 (22.5)	1138 (29.2)	2452 (19.5)
80-89 years	1871 (43.8)	728 (18.7)	1239 (9.8)
≥90 years	1091 (25.5)	139 (3.6)	131 (1.0)
Sex			
Female	2043 (47.8)	1421 (36.5)	5151 (40.9)
Male	2232 (52.2)	2476 (64.5)	7441 (59.1)
Comorbidities			
Asthma	424 (9.9)	440 (11.3)	1488 (11.8)
Cancer	587 (13.7)	541 (13.9)	613 (4.9)
Cardiac/cerebrovascular	2655 (62.1)	1339 (34.4)	2577 (20.5)
Chronic kidney disease	1092 (25.5)	537 (13.8)	787 (6.2)
Chronic liver disease	123 (2.9)	136 (3.5)	298 (2.4)
Chronic lung disease	895 (20.9)	521 (13.4)	974 (7.7)
Diabetes	1328 (31.1)	1080 (27.7)	2487 (19.8)
Hypertension	3120 (73.0)	2103 (54.0)	4815 (38.2)
Immunosuppression	655 (15.3)	692 (17.8)	1347 (10.7)
Neurology/Dementia	1069 (25.0)	263 (6.7)	501 (4.0)
Obesity	292 (6.8)	434 (11.1)	1560 (12.4)
Psychiatric	1111 (26.0)	900 (23.1)	3344 (26.6)
Care services	2657 (62.2)	893 (22.9)	974 (7.7)
First wave	1344 (31.4)	1283 (32.9)	2340 (18.6)
Education level			
Primary education	1440 (33.7)	1028 (26.4)	2793 (22.2)
Secondary education	1663 (38.9)	1603 (41.1)	5090 (40.4)
Tertiary education	899 (21.0)	1097 (28.1)	4166 (33.1)
Missing	273 (6.4)	169 (4.3)	543 (4.3)
Region of birth			
Sweden	2994 (70.0)	2216 (56.9)	6935 (55.1)
Northwestern Europe and Northern America	544 (12.7)	330 (8.5)	749 (5.9)
Southern Europe	131 (3.1)	165 (4.2)	477 (3.8)
Eastern Europe	102 (2.4)	120 (3.1)	435 (3.5)
Western and Central Asia	315 (7.4)	503 (12.9)	1862 (14.8)
East Asia and Oceania	72 (1.7)	235 (6.0)	936 (7.4)
Africa	60 (1.4)	175 (4.5)	630 (5.0)
Latin America and the Caribbean	50 (1.2)	145 (3.7)	508 (4.0)
Missing	7 (0.2)	8 (0.2)	60 (0.5)
Household composition			
Single-person household	2172 (50.8)	1215 (31.2)	3133 (24.9)
Multi-person household	2079 (48.6)	2659 (68.2)	9298 (73.8)
Missing	24 (0.6)	23 (0.6)	161 (1.3)

Note: Values are shown as counts with percentages representing proportions in parenthesis.

ESM Table 2. Interaction tests of socioeconomic factors with NEWS, age ≥75 years and calendar time

Interaction terms	*P-value*
Education level + NEWS	0.1500
Region of birth + NEWS	0.1340
Household composition + NEWS	0.0009
Education level + Age 75	0.0098
Region of birth + Age 75	0.1151
Household composition + Age 75	<0.0001
Education level + calendar time	0.2448
Region of birth + calendar time	0.0010
Household composition + calendar time	0.0004

Abbreviations: NEWS = National Early Warning Score
Note: NEWS is a binary variable indicating if NEWS ≥ 7. Age 75 is a binary variable indicating if age ≥ 75 years. Calendar time was modelled using restricted cubic splines, with knots placed as described in the Methods section.

ESM Figure 1. Stratified analyses following identified interactions between socioeconomic factors and NEWS and age ≥ 75 years.

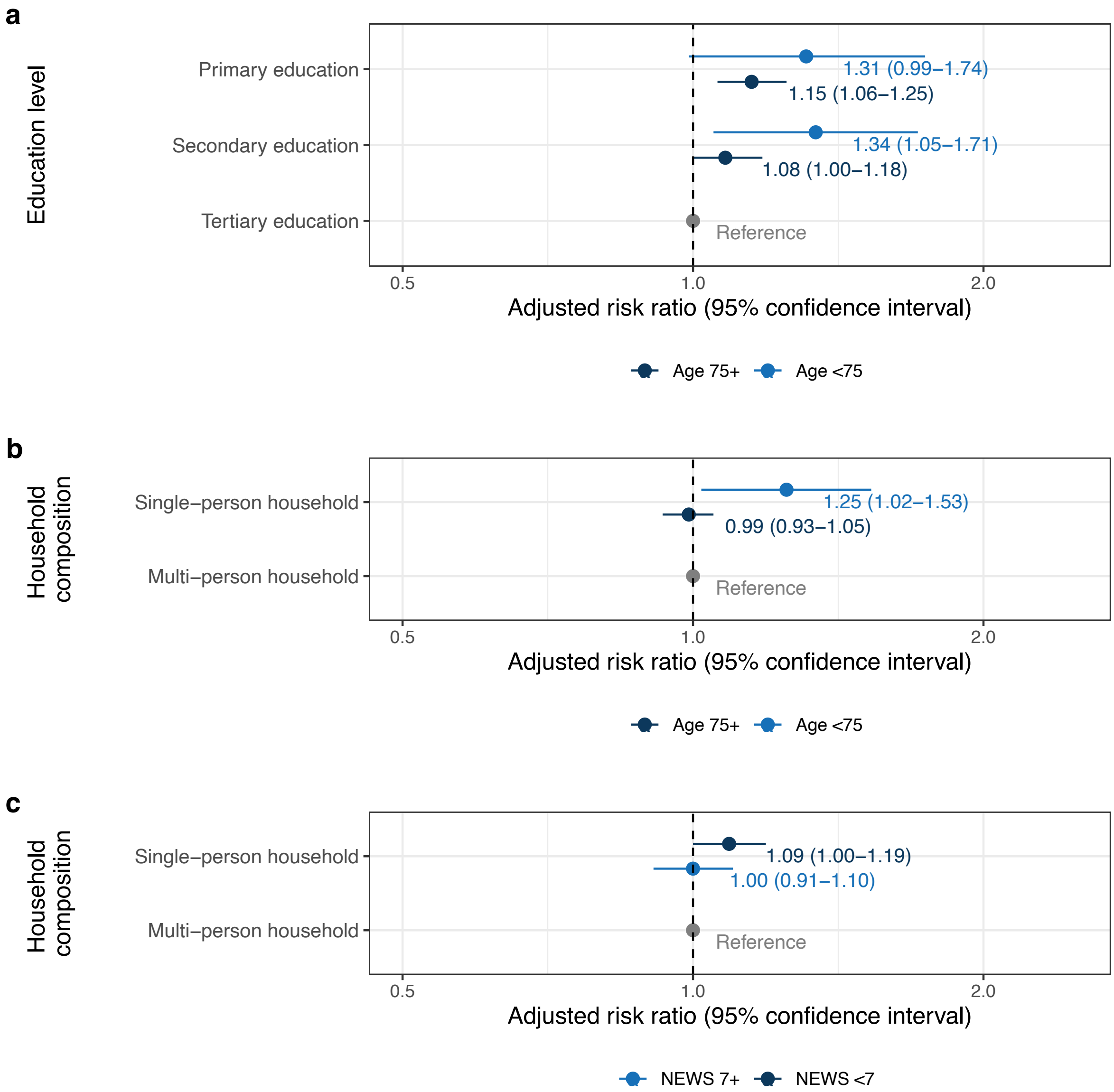

Note: Panel a shows the adjusted risk ratio of categories of education level stratified by age ≥ 75 years vs age < 75 years, adjusted for region of birth, household composition, age, sex, comorbidities, care services, calendar time, hospital, and ICU occupancy. Panel b shows the adjusted risk ratio of categories of household composition stratified by age ≥ 75 years vs age < 75 years, adjusted for education level, region of birth, age, sex, comorbidities, care services, calendar time, hospital, and ICU occupancy. Panel c shows the adjusted risk ratio of categories of household composition stratified by NEWS ≥ 7 points vs NEWS < 7 points, adjusted for education level, region of birth, age, sex, comorbidities, care services, calendar time, hospital, and ICU occupancy.
