# Supplementary material for: Socioeconomic factors and non-admission to the intensive care unit decisions in hospitalised COVID-19 patients: a retrospective cohort study in Stockholm, Sweden
Source: BMJ Open. 2026 Aug 27;16(8):e120447. doi: 10.1136/bmjopen-2026-120447 (PMC13536059; doi:10.1136/bmjopen-2026-120447)
Supplement: Supplementary file 2 [file bmjopen-2026-120447.reviewer_comments.pdf]

PEER REVIEW HISTORY

BMJ Open publishes all reviews undertaken for accepted manuscripts. Reviewers are asked to complete a checklist review form and are provided with free text boxes to elaborate on their assessment. These free text comments are reproduced below.

ARTICLE DETAILS

Title (Provisional)

Socioeconomic factors and non-admission to the intensive care unit decisions in hospitalised COVID-19 patients: a retrospective cohort study in Stockholm, Sweden

Authors

Sotoodeh, Adonis; Hedberg, Pontus; Zetterqvist, Johan; Mårtensson, Johan; Naucler, Pontus

---

VERSION 1 - REVIEW

**Reviewer**	**1**
**Name**	**Jaakkimainen, R. Liisa**
**Affiliation**	**Institute for Clinical Evaluative Sciences**
**Date**	**29-May-2026**
**COI**	**None**

This is a well written and thoughtfully conducted record linkage study examining the association of socioeconomic factors to non-admission to the ICU amongst patients admitted to hospital for COVID 19. My comments should be considered minor.

What proportion of patients are not admitted through the emergency department in Sweden? Just to have a comparison with other countries where the vast majority of admissions come via the emergency department.

20,764 COVID admissions seem lower compared to other countries? Is this correct?

What would be the impact of a large % of admissions having no documentation of recorded decision? The introduction mentioned standard protocols for making the decision about an ICU admission. What are the specifics of these protocols and wouldn't this be included?

Have SES factors been examined in related to the decision to admit from the community to the hospital? For example, in other jurisdictions, those with lower SES compared to higher got admitted to the hospital for COVID 19. If a higher proportion of lower educated or socially disadvantaged people get admitted, this may dampen your association with the decision not to admit to the ICU (though it still may have a positive association).

What were the most responsible diagnoses for these hospital admissions?

For people born outside of Sweden was there any indication how long they lived in Sweden?

**Reviewer**	**2**
**Name**	**Biundo, Eliana**
**Affiliation**	**GSK Belgium**
**Date**	**01-Jun-2026**
**COI**	**None**

Very interesting piece of research. The aim of the paper is to investigate the relationship between socio economic status and ICU admissions. From the methods and results presented it remains unclear on the percentage of participants that did receive an ICU admission and their characteristics. It is difficult to assess association of social determinants of health in the decision of non admission without understanding the population characteristics of those that were admitted to the ICU. The authors correctly note in the introduction the pressure on ICU units during the peak of the pandemic. An analysis by year or COVID peak should be performed to better understand the relationship between ICU occupancy and decision on non admission. It might be valuable to also understand what was ICU occupancy in the study hospitals during the study period. This would allow to better put into perspective if the findings are truly associated with socioeconomic status or if there are additional confounding factors not accounted for in the analysis. Finally, results section is quite short and would benefit from some additional analysis descriptions.

VERSION 1 - AUTHOR RESPONSE

Reviewer 1

This is a well written and thoughtfully conducted record linkage study examining the association of socioeconomic factors to non-admission to the ICU amongst patients admitted to hospital for COVID 19. My comments should be considered minor. What proportion of patients are not admitted through the emergency department in Sweden? Just to have a comparison with other countries where the vast majority of admissions come via the emergency department.

Thank you for your careful assessment of our manuscript and your kind feedback. The proportion of patients admitted through the emergency department varies between conditions, but for acute respiratory infections it is high. In our setting, admission via the emergency department was an inclusion criterion; this captured 20 764 of 22 501 hospitalised patients (94.2%) with Covid-19, indicating that only a small minority were admitted through other routes. We have rephrased a sentence in the Methods section to clarify this.

"Only patients admitted through the emergency department were included, corresponding to approximately 94% of those who otherwise met the inclusion criteria"

20,764 COVID admissions seem lower compared to other countries? Is this correct?

Thank you for this observation. The number of admissions reflects the regional scope of our study, which was restricted to five out of the six acute care hospitals in Region Stockholm rather than the whole of Sweden. For context, national statistics from the Swedish National Board of Health and Welfare report approximately 96 500 COVID-19 hospitalisations across Sweden (https://www.socialstyrelsen.se/en/statistics-and-data/statistics/statistics-subjects/statistics-on-covid-19/, accessed 260611); as Region Stockholm comprises roughly one quarter of the national population, and our cohort additionally reflects the applied inclusion and exclusion criteria, the number of patients is consistent with the expected regional share. We therefore believe the number is in line with national data once the regional scope and study population restrictions are taken into account.

What would be the impact of a large % of admissions having no documentation of recorded decision?

We appreciate this important point. In Sweden, the physician responsible for a patient's care is required to thoroughly document any decision on treatment limitations, including decisions on non-admission to the intensive care unit. The absence of such a limitation can therefore be interpreted as the absence of such a limitation, meaning these patients were eligible for ICU admission without restriction. To assess the robustness of this assumption, we performed a sensitivity analysis in which a decision was imputed for patients without a documented decision. The results were consistent with our main analysis, indicating that our findings are not sensitive to how undocumented decisions were handled.

The introduction mentioned standard protocols for making the decision about an ICU admission. What are the specifics of these protocols and wouldn't this be included?

Thank you for raising this. In our setting there is no formal protocol or guideline specifying fixed criteria for the decision on ICU admission; the determination is made by the treating physician based on an individual clinical assessment of each patient. There are therefore no standardised protocol criteria that could have been incorporated as variables in the analysis.

Have SES factors been examined in related to the decision to admit from the community to the hospital? For example, in other jurisdictions, those with lower SES compared to higher got admitted to the hospital for COVID 19. If a higher proportion of lower educated or socially disadvantaged people get admitted, this may dampen your association with the decision not to admit to the ICU (though it still may have a positive association).

Thank you for this thoughtful point. We agree that the pathway from the community to hospital may be patterned by socioeconomic factors, and that our cohort, which by design begins at hospital admission, cannot examine this earlier step. We would note, however, that this patterning is most likely to arise in health-care-seeking behaviour and presentation to hospital, rather than in the decision to admit from the emergency department, which we expect to be primarily clinically driven. As the reviewer notes, if individuals of lower socioeconomic position are more likely to present and to be admitted, this selection would, if

anything, be expected to attenuate association on non-admission to the ICU, rather than create a false positive association. The associations we report may therefore represent a conservative estimate. We have added a sentence to the Discussion acknowledging this limitation.

"Fourthly, our cohort began at hospital admission, so we could not determine whether socioeconomic factors influenced presentation to hospital from the community. While we would not anticipate socioeconomic influences on the emergency department's clinical admission decision, any patterning in care-seeking behaviour, if present, would be expected to weaken rather than inflate the observed associations."

Reviewer 2

Very interesting piece of research. The aim of the paper is to investigate the relationship between socio economic status and ICU admissions. From the methods and results presented it remains unclear on the percentage of participants that did receive an ICU admission and their characteristics. It is difficult to assess association of social determinants of health in the decision of non-admission without understanding the population characteristics of those that were admitted to the ICU.

Thank you for the kind assessment of our work and for the opportunity to clarify the scope of our study. We would respectfully clarify that our objective was not to investigate which patients were ultimately treated in the ICU, but rather which patients received a decision that they would not be admitted to the ICU should the need arise. Patients without such a decision were considered eligible for ICU admission, irrespective of whether they subsequently deteriorated to the point of requiring it. We would therefore gently note that the characteristics of patients admitted to the ICU reflect subsequent clinical course rather than the decision under study, and that analysing them would risk conflating the decision itself with downstream clinical events that fall outside our research question. We hope this clarifies the scope of our analysis and we are grateful for the opportunity to make it explicit.

The authors correctly note in the introduction the pressure on ICU units during the peak of the pandemic. An analysis by year or COVID peak should be performed to better understand the relationship between ICU occupancy and decision on non admission. It might be valuable to also understand what was ICU occupancy in the study hospitals during the study period. This would allow to better put into perspective if the findings are truly associated with socioeconomic status or if there are additional confounding factors not accounted for in the analysis.

Thank you for this thoughtful comment. We fully agree that ICU occupancy is an important factor in understanding decisions on non-admission to the ICU during the pandemic, and it was included as a covariate in our adjusted models, thereby accounting for the variation in pressure on the ICU across the study period, including peaks and troughs. To clarify, ICU occupancy was assessed using a 7-day rolling average number of ICU patients with COVID-19 for each calendar day, including the date of admission, capturing fluctuations in ICU pressure across the study period, including pandemic peaks.

We have also examined the relationship between ICU and hospital occupancy and non-admission decisions in greater depth in a related study from the same setting, which describes

occupancy levels in these hospitals across the study period and found that the risk of non-admission decisions increased with higher COVID-19 ICU and hospital occupancy. We believe these analyses together indicate that the socioeconomic associations we report are not explained by occupancy or by temporal variations in ICU pressure. We hope this satisfactorily addresses the question.

We have made the following clarification to the manuscript in the Methods section:

ICU occupancy was assessed using a 7-day rolling average number of ICU patients with COVID-19 for each calendar day, including the date of admission, capturing fluctuations in ICU pressure across the study period, including pandemic peaks."

Finally, results section is quite short and would benefit from some additional analysis descriptions.

Thank you for this suggestion. We have expanded the results section to provide additional detail, including the addition of p-values alongside the risk ratios and 95% confidence intervals for all analyses. In addition, in response to the previous comment, we extended our interaction analyses to also test for interaction with calendar time, alongside the previously reported interactions with NEWS ≥7 points and age ≥75 years. We hope this provides a fuller account of our findings. We have made the following addition to the manuscript:

Methods: *"Lastly, we tested for interaction between the socioeconomic variables and the dichotomous variables NEWS ≥ 7 and age ≥ 75 years, as well as calendar time (modelled using restricted cubic splines), using Wald tests."*

Results: "*In interaction analyses, interactions were found between household composition with NEWS ≥7 points (p = 0.0009), education level with age ≥75 years (p = 0.0098), household composition with age ≥75 years (p = <0.0001), region of birth with calendar time (p = 0.0010), and household composition with calendar time (p = 0.0004) (ESM Table 2). Stratified analyses for the categorical effect modifiers (age ≥75 years and NEWS ≥7 points) are provided in the supplementary material (ESM Figure 1). As calendar time was modelled using restricted cubic splines, the corresponding interactions could not be summarised as stratum-specific odds ratios and are therefore not presented as stratified plots."*

Discussion: *"Notably, household composition showed significant interactions with NEWS ≥7, age ≥ 75 years, and calendar time, suggesting that its effect may not be uniform across time periods or patient severity and age groups, which could further contribute to the absence of a consistent overall association."*

VERSION 2 - REVIEW

**Reviewer**	**1**
**Name**	**Jaakkimainen, R. Liisa**
**Affiliation**	**Institute for Clinical Evaluative Sciences**
**Date**	**13-Aug-2026**

COI

The authors responded well to the comments from the previous review.

I have no additional comments.

**Reviewer**	**2**
**Name**	**Biundo, Eliana**
**Affiliation**	**GSK Belgium**
**Date**	**29-Jul-2026**
**COI**	

---

thank you for addressing the feedback received
